# Prefilled pen versus prefilled syringe: a pilot study evaluating two different methods of methotrexate subcutaneous injection in patients with JIA

**DOI:** 10.1186/s12969-020-00455-4

**Published:** 2020-08-12

**Authors:** Justyna Roszkiewicz, Zbigniew Swacha, Elżbieta Smolewska

**Affiliations:** 1 Department of Paediatric Cardiology and Rheumatology, Medical University of Lodz, Sporna 36/50, 91-738 Lodz, Poland; 2 Clinic of Dermatology, Military Medical Institute, Warsaw, Poland

**Keywords:** Methotrexate, Juvenile idiopathic arthritis, Autoinjector, Pen, Prefilled syringe

## Abstract

**Background:**

Methotrexate is the most commonly used disease-modifying antirheumatic drug recommended in the treatment of juvenile idiopathic arthritis. It can be administered orally or subcutaneously, the latter method is associated with fewer side effects and higher drug bioavailability. Nevertheless, the pain associated with injection is a considerable drawback of this treatment option in the pediatric population. Currently, there are two single-use subcutaneous injection devices available: the prefilled syringe and the prefilled pen. This prospective, two-sequence crossover study aimed to compare ease of use, frequency of therapy side effects, injection-site pain and parent/patient preference of those methotrexate parenteral delivery systems.

**Methods:**

Twenty-three patients with juvenile idiopathic arthritis, already treated with subcutaneous methotrexate in the form of prefilled syringe in the period October 2018 – April 2019 completed a questionnaire evaluating their experience with this device. Subsequently, children received a one-month supply of pen autoinjector and completed the same questionnaire, regarding their experience with the new methotrexate delivery system. If the patient was not performing the injections himself the questionnaires were completed by the caregiver administrating MTX. The results obtained in both questionnaires were compared using the Wilcoxon matched-pairs signed-rank test.

**Results:**

82,6% patients and their caregivers voted for the prefilled pen as their preferred method of subcutaneous methotrexate administration. Moreover, the injection with the prefilled pen was reported as less painful in comparison to the prefilled syringe (*p* < 0.01). Side effects of methotrexate were less pronounced after the prefilled pen treatment, this difference was most prominent regarding gastrointestinal adverse events associated with the injection (*p* < 0.01).

**Conclusion:**

Administration of methotrexate using the pen device is a promising way of subcutaneous methotrexate delivery in children with juvenile idiopathic arthritis, as the injection is less painful and associated with fewer side effects.

## Background

Juvenile idiopathic arthritis (JIA) is the most common chronic rheumatic disease in children, with an estimated prevalence between 16 and 150 per 100,000 [1]. It is defined by the International League of Associations for Rheumatology (ILAR) as arthritis of an unknown etiology that persists for at least 6 weeks in children under the age of sixteen [2]. Although biologic agents are increasingly used in the management of this condition, methotrexate (MTX) remains the mainstay of JIA treatment [3, 4]. MTX is administered weekly at a dose of 10–15 mg/m^2^ either via oral or parenteral route [5]. The bioavailability of MTX is about 15% higher after subcutaneous administration than after oral intake, leading to the improvement of treatment efficacy [6, 7]. Moreover, the most common side effect of MTX therapy, gastrointestinal toxicity, is less pronounced after the MTX injection [8]. Nevertheless, pain and stress associated with subcutaneous injections are a significant drawback of this treatment, particularly prominent in younger patients. Subcutaneous MTX may be administered via two devices: the prefilled syringe or, recently introduced to the market, the pen autoinjector. The latter device was preferred by patients with rheumatoid arthritis (RA) with regards to overall satisfaction and ease of use [9]. The aim of this study was to assess the experience of patients with JIA and their caregivers who used both the prefilled syringes and the prefilled pens, concerning parents’ and patients’ preference, usability, and tolerability outcomes.

## Methods

### Patients

This was a prospective, two-sequence crossover study performed in one pediatric rheumatology centre in Poland. Patients were eligible for the study if they were between 2 and 18 years of age and had the diagnosis of JIA made according to ILAR criteria [2]. Moreover, the ongoing subcutaneous MTX therapy using the prefilled syringe (dose 10–15 mg/m2) was required to be included in the study group. Exclusion criteria comprised previous treatment with the autoinjecting device and the presence of contraindications to continuing MTX therapy at the baseline of the study.

### Study intervention

Patients eligible for the study received a questionnaire in which they have assessed their experience with the preceding prefilled syringe (Metex®; Medac GmbH) treatment. The questionnaire consisted of 3 parts:

- Part 1: 7 questions regarding the use of device (ease of use, convenience of injection operation, confidence regarding the device proper use, the device characteristics), answered in the Likert manner;
- Part 2: evaluation of pain associated with the injection by the patient using Faces – Pain Scale – Revised (FPS-R [10]) and Face, Legs, Activity, Cry, Consolability scale (FLACC [11]) assessing the level of pain based on the changes in the child’s behavior;
- Part 3: assessment of treatment side effects (local skin reactions, nausea, vomiting, abdominal pain) – multiple-choice questions (a- side effect absent, b-present in < 50% of injections, c-present in > 50% of injections d- present in 100% injections e-present in 100% of injections and very severe).

If the patient was not giving the MTX injections himself, the questionnaire was completed by the caregiver, with the exception for the question assessing the pain associated with injection using the FPS-R [10]. In patients administrating MTX by themselves FLACC [11] value was assessed by the caregiver supervising the injection. Subsequently, patients received 4-weeks supply of the prefilled pen (Metex Pen®; Medac GmbH), at the same MTX dose as used during the prefilled syringe treatment. Before the first injection, patients and their caregivers were made familiar with the new injection system by the study nurse. After the one-month period of prefilled pen treatment patients received the questionnaire again, this time evaluating their experience with the new drug delivery system. In the second questionnaire patients were also required to answer the question about their overall preference of subcutaneous MTX delivery method.

### Study endpoints

The primary end point of the study was the number of patients preferring the MTX prefilled pen over the prefilled syringe after 1 month of treatment. Secondary end points included a comparison of the self-injection experience of the patients after each treatment period and frequency of treatment side effects.

### Statistical analysis

Results from the questionnaire subcategories 1 and 3 were transferred to 0–10 numeric scale, in which 0 was associated with the worst and 10 with the best patient experience. Outcomes of FLACC were assessed using the calculator available online [12]. In the case of multiple-choice questions, the following converter applied: answer a − 10, b-7.5, c-5, d-2.5, e-0. The median values and interquartile ranges (IQR) achieved in each subcategory were calculated using the simple descriptive statistics. The normality of distribution was checked using the Kolmogorov-Smirnov (K-S) test. Subsequently, the results obtained in both questionnaires were compared using the Wilcoxon matched-pairs signed-rank test. All statistical calculations were made using Statistica 13.1 software (TIBCO, Palo Alto, CA, USA).

## Results

### Study group characteristics

The study group was composed of 23 patients with JIA, 17 girls and 6 boys with the mean age 11.7 years. The mean time interval between the diagnosis of JIA was 4.23 year, with a minimum of 3 months and a maximum of 13.5 years. The mean time of subcutaneous MTX treatment equaled 18.52 months, with a minimum of 3 months and a maximum of 5 years. Twelve patients (52.2%) from the study group were diagnosed with oligoarticular JIA, 6 (26.1%) with polyarticular seronegative JIA, 4 (17.4%) with enthesitis-related arthritis (ERA) and 1 (4.3%) with systemic subtype of JIA. Sixteen patients (69.5%) were receiving MTX at the dose of 15 mg, 4 (17.4%) were treated with 10 mg and 3 (13.1%) with 20 mg. Only 3 (13.1%) patients were performing the injections by themselves.

### Overall patient preference

The overall median values of patient satisfaction equaled 5/10 (IQR 5.0) for the prefilled syringe and 10/10 (IQR 2.0) for the prefilled pen (*p* < 0.01). 19/23 patients or caregivers (82.6%) voted for the prefilled pen as their preferred method of subcutaneous MTX administration.

### Ease of device use

**Fig. 1:**
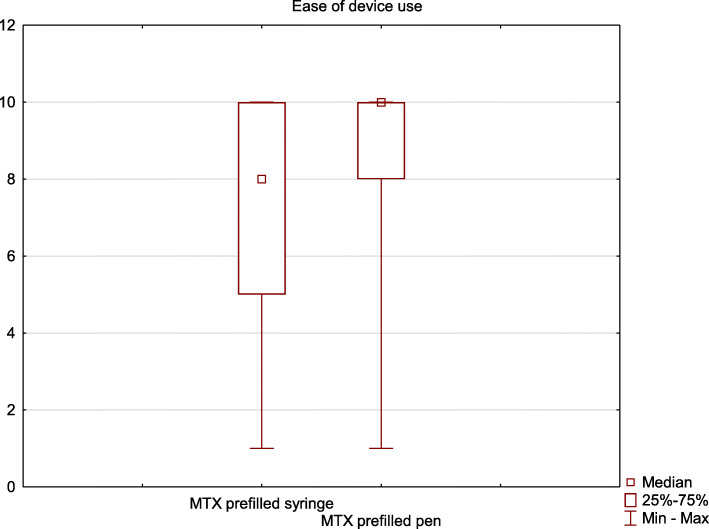
Comparison of methotrexate prefilled pen and prefilled syringe with regard to ease of the device use (0 - worst possible experience, 10 - best possible experience)

Table 1 summarizes the results obtained in this section. Overall, despite the patients used the prefilled syringe for a considerably longer period than the pen, the latter device was ranked as significantly easier to use (*p* < 0.01) (Fig.1).

**Table 1:** Comparison of results obtained by methotrexate prefilled pen and prefilled syringe in the “Ease of use” section of the questionnaire

Question	Device	Median value	Interquartile range	*P*-value
The injection is easy to perform	Prefilled syringe	8.0	5.0	0.06
	Prefilled pen	10.0	2.0	
I do not have any problems with performing the injection	Prefilled syringe	8.0	7.0	**0.04**
	Prefilled pen	10.0	2.0	
The injection system lies comfortably and secure in the hand during the injection	Prefilled syringe	8.0	4.0	**0.012**
	Prefilled pen	10.0	2.0	
It was easy to learn how to perform the injection correctly	Prefilled syringe	8.0	6.0	**0.008**
	Prefilled pen	10.0	1.0	
**Overall**	Prefilled syringe	8.0	5.0	**0.00001**
	Prefilled pen	10.0	2.0

### Self-confidence regarding the device proper use

**Fig. 2:**
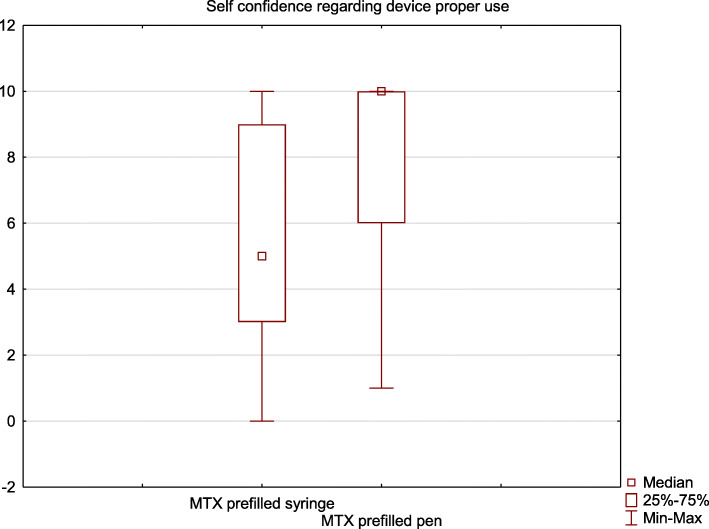
Results obtained by the methotrexate prefilled pen and prefilled syringe in “Self-confidence regarding the device proper use” questionnaire section (0- worst possible experience, 10 - best possible experience)

The caregivers’ confidence regarding the device proper use was significantly higher after the period of prefilled pen treatment, what corresponded to a lower level of stress associated with MTX administration (*p* < 0.01) (Fig.2). The detailed results are listed in Table 2.

**Table 2:** Detailed comparison of results obtained by both devices in “Self-confidence regarding the device proper use” section of the questionnaire

Question	Device	Median value	Interquartile range	*P*-value
I don’t have any objections against performing the injection with this device	Prefilled syringe	8.0	5.0	0.028
	Prefilled pen	10.0	2.0	
I am not stressed when the day of MTX administration comes	Prefilled syringe	3.0	6.0	**0.013**
	Prefilled pen	9.0	7.0	
I feel confident performing the injection on my own	Prefilled syringe	5.0	7.0	**0.004**
	Prefilled pen	10.0	5.0	
**Overall**	Prefilled syringe	5.0	6.0	**0.00002**
	Prefilled pen	10.0	4.0	

### Pain associated with the injection

**Fig. 3:**
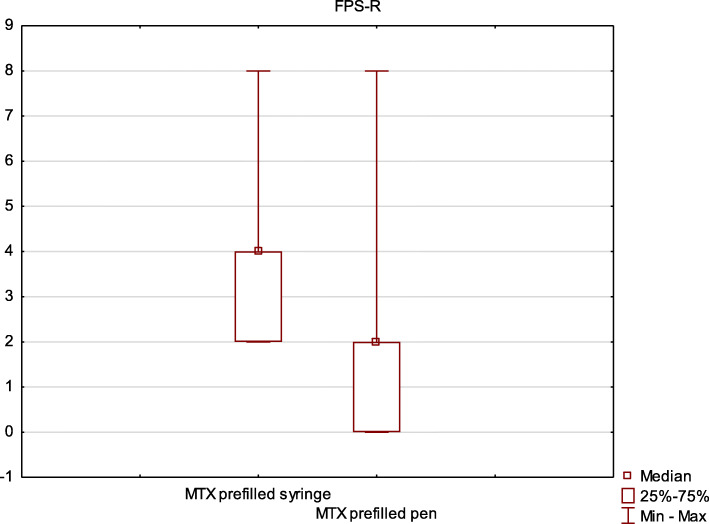
The comparison of pain associated with the methotrexate injection using prefilled pen and prefilled syringe - results obtained using the Faces Pain Scale – Revised (FPS-R). 0 - no pain, 10 - most severe pain

**Fig. 4:**
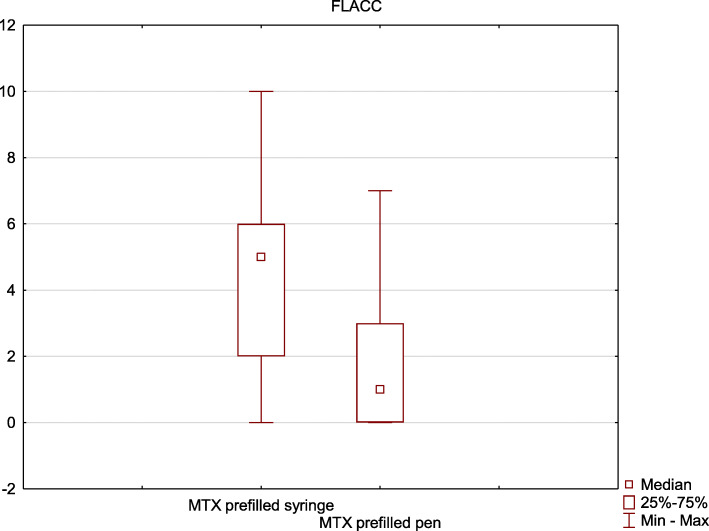
The comparison of pain associated with the methotrexate injection using prefilled pen and prefilled syringe – results obtained using the Face, Leg, Activity, Cry, Consolability (FLACC) scale. 0 - no pain, 10 - most severe pain

The level of pain was significantly lower after the injection performed with the MTX prefilled pen. This observation concerned both the level of pain assessed by patients (FPS-R median 4.0 vs 2.0, *p* = 0.001 (Fig.3)) and their caregivers (FLACC median 5.0 vs 1.0, *p* = 0.0004 (Fig.4)).

### Side effects of subcutaneous MTX treatment

In general, the side effects of MTX treatment were observed in 16/23 patients (69.5%) during the prefilled syringe treatment and in 8/23 (34.7%) treated with MTX prefilled pen. Local symptoms associated with injection were infrequent and there was no statistically significant difference between both devices (Table 3). Nevertheless, remarkably more patients treated with prefilled syringe experienced nausea, vomiting (*p* = 0.007) and abdominal pain (*p* = 0.003).

**Table 3:** Frequency of side effects associated with subcutaneous MTX treatment – comparison of the prefilled pen and prefilled syringe

	Side effects of MTX treatment							
Side effect	Device	Absent	Present in < 50% of injections	Present in > 50% of injections	Always present	Always present and very severe	Number of points	*P*-value
Local redness of injection site	Prefilled syringe	20 (87%)	2 (8.7%)	1 (4.3%)	0	0	220	0.42
	Prefilled pen	21 (91.3%)	2 (8.7%)	0	0	0	215	
Swelling of the injection site	Prefilled syringe	21 (91.3%)	2 (8.7%)	0	0	0	225	0.18
	Prefilled pen	22 (95.7%)	1 (4.3%)	0	0	0	227.5	
Hematoma of the injection site	Prefilled syringe	21 (91.3%)	1 (4.3%)	1 (4.3%)	0	0	222.5	0.18
	Prefilled pen	23 (100%)	0	0	0	0	230	
Itching of the injection site	Prefilled syringe	20 (87%)	1 (4.3%)	2 (8.7%)	0	0	217.5	0.11
	Prefilled pen	23 (100%)	0	0	0	0	230	
Nausea and vomiting associated with MTX administration	Prefilled syringe	9 (39,1%)	3 (13,04%)	5 (21,7%)	3 (13,04%)	3 (13,04%)	145	**0.007**
	Prefilled pen	17 (73.9%)	3 (13,04%)	1 (4,3%)	0	2 (8,7%)	197,5	
Abdominal pain associated with MTX administration	Prefilled syringe	8 (34,8%)	4 (17.4%)	4 (17.4%)	6 (26,1%)	1 (4.3%)	160	**0.003**
	Prefilled pen	17 (73.9%)	2 (8.7%)	3 (13.04%)	1 (4.3%)	0	217.5	

## Discussion

Up to our best knowledge, currently there are no studies assessing the features of drug delivery systems important for patients with JIA and their caregivers. Studies conducted among individuals with RA showed the preference for subcutaneous injections in comparison to intravenous infusions and tendency to select ready to use drug delivery systems, with a high preference for pen autoinjectors [13–17]. Moreover, the risk of side effects associated with treatment method was not neglectable [18]. Currently, MTX is available in Poland in two forms: oral pills and subcutaneous prefilled syringes. The parenteral way of MTX administration is preferred by clinicians, as it provides dependable efficacy, predictable bioavailability, sustained clinical outcomes, and lower risk of adverse effects [19, 20]. Nevertheless, the caregivers are frequently reluctant to this method of treatment, usually due to the pain associated with injection and low level of self-confidence regarding the device proper use. The newly developed prefilled pen may become a long-awaited solution to this problematic issue.

In our study, we have assessed the overall patients and caregivers preference for MTX pen as 82.6%. Despite the small size of the study population, the results are comparable to those obtained in patients with RA, where the preference for pen reached 75% [9]. Interestingly, despite our patients received the prefilled syringe treatment for a considerably longer period than the prefilled pen, their caregivers have evaluated the latter device as easier to use (*p* < 0.01). Moreover, it corresponded to the higher level of their self-confidence regarding the device proper use (*p* < 0.01).

Pain associated with the drug administration is a significant drawback of treatment, especially pronounced in the pediatric population. In our study, the injection performed with the prefilled pen was reported as less painful both by patients and their caregivers.) Those results are contradictory to the outcomes of the study performed in RA patients, although those findings are not directly comparable as different measures (Self-injection Assessment Questionnaire [21] in RA patients) were applied when assessing this parameter.

The frequency of gastrointestinal side effects during MTX pen and syringe treatment was not evaluated in the previous studies. In our patients’ nausea, vomiting and abdominal pain were significantly less pronounced during the treatment with MTX pen (*p* < 0.01), although the dosage of the drug was not changed during the examination period and drug pharmacokinetic properties were assessed as comparable during treatment with similar devices in previous studies [22]. This finding implies that the vast majority of gastrointestinal MTX adverse effects present in the pediatric population may not be the effect of drug toxicity itself, but the result of a stress reaction to the injection process. The MTX pen is equipped with a special needle cover system, which was constructed in order to diminish the risk of caregivers’ needlestick injury. What is more, the invisibility of the needle during the injection may lead to a lower level of stress and pain associated with the drug administration in the children with JIA [23].

Although very promising, the results of our study must be interpreted with caution and a number of limitations should be borne in mind. To begin with, it was a pilot study, conducted on relatively small study group with low representation of children administrating MTX by themselves (3/23, 13.1%). In consequence, the overall preference of MTX autoinjector and frequency of treatment side effects was assessed by the caregivers in 20/23 (86.9%) children and therefore may not reflect the actual opinion of patients themselves. However, we consider this limitation as unlikely to influence our results, as pain level which was assessed both by children (FPS-R) and their caregivers (FLACC) was similar, suggesting that the observation of a caregiver reflects well the opinion of their child. Moreover, the JIA patients enrolled to this project were already treated with subcutaneous MTX in the form of prefilled syringe. Thus, the impact of new autoinjector novelty effect on our results can not be excluded. Future studies, including larger group of subcutaneous MTX-naive JIA patient and enriched with the assessment of the injection process by both patient and caregiver conducted in the crossover design are needed to verify our findings.

## Conclusion

The prefilled pen is a promising method of MTX parenteral delivery in children with JIA, as the injection is less painful and associated with fewer side effects that the one performed with the prefilled syringe. Those results may be applied in the everyday practice of pediatric rheumatologist in the future, as the optimal choice of drug delivery system may obviously result in improved patients’ compliance and consequently in lower disease activity. Future studies in this area including larger cohorts of subcutaneous MTX- naive patients performed in a crossover design are encouraged to verify our findings.

## Data Availability

The data analyzed during this study are included in tables attached to this article.

## Abbreviations

JIA: Juvenile Idiopathic Arthritis
ILAR: International League of Associations for Rheumatology
MTX: Methotrexate
RA: Rheumatoid Arthritis
FPS-R: Faces Pain Scale–Revised
FLACC: Face, Legs, Activity, Cry, Consolability
IQR: Interquartile Range
K-S test: Kolmogorov-Smirnov test
ERA: Enthesitis-Related Arthritis
